# Effects of thermal and nonthermal processing technology on the quality of red sour soup after storage

**DOI:** 10.1002/fsn3.2366

**Published:** 2021-06-05

**Authors:** He Yangbo, Li Yongfu, Luo Xingbang, Li Guolin, Duan Zhaoyan, Chen Chaojun

**Affiliations:** ^1^ Insititute of Integrated Agricultural Development Guizhou Academy of Agricultural Science Guiyang China; ^2^ Jinsha Guan Xiang Fang Seasoning Food Co., Ltd. Bijie China; ^3^ Guizhou Jinnong Radiation Technology Co., Ltd. Guiyang China; ^4^ Guizhou Institute of Biological Technology Guiyang China

**Keywords:** red sour soup, thermal and nonthermal preservation, microflora, sensory analysis

## Abstract

In the present study, we investigated the effects of thermal preservation, such as pasteurization, and nonthermal preservation, including irradiation, sodium dehydroacetate (SDHA), and nisin, on the quality of red sour soup after storage. Single‐factor experiments were used to optimize the parameters of different processing technologies, and the best irradiation dose and heating temperature were 4 kGy and 85℃, respectively. The optimal additive amounts of SDHA and nisin were 150 mg/500 g. During the shelf storage experiment, prepared red sour soup was stored at room temperature in the glass bottles, and further analyses were carried out up to 5 weeks of storage. The quality of red sour soup was evaluated by microflora and sensory analysis. The results showed that *Lactobacillus*, *Streptomyces, Pediococcus*, *Pichia*, *Kazachstania*, and *Candida* were the main microorganisms in all samples, and there were no harmful microorganisms. The sensorial attributes were observed, including different parameters, such as odor, organic acid content, color, taste, texture, apparent viscosity, and thixotropy. All of the data showed that the irradiated groups were more dramatically changed compared with the other groups, while these changes did not directly affect the sensory quality of the products. Consequently, irradiation could be used as an ideal quality preservation method for the red sour soup to reduce the impact of heat treatment and chemical additives on the quality of characteristic food.

## Graphical abstract

This article compared the effect of different preservation methods on the quality of red sour soup and provided more schemes for the storage and preservation of related fermentation products.

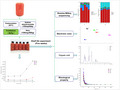

## 1 INTRODUCTION

Red sour soup is a type of semisolid or liquid condiment, which is made up of tomato and/or red pepper as the main raw materials, with the supplementation of certain proportions of ginger, edible salt, liquor, and other auxiliary materials (CNFIA, 2020). Although the raw materials of red sour soup are similar to ketchup, they are different in processing technology, cooking method, and sensory experience. Red sour soup has been developed into one of the three hot pot bottoms in China, together with spicy and clear soup (Zeng et al., 2014). However, due to the high moisture content, rich nutrition, and complex microbial flora, the shelf‐life deterioration issues, such as spoilage, mildew, and flatulence, have restricted the development of the red sour soup industry (Lu et al., 2019; Zou, 2015; Wei 2016; Zhang, 2011).

At present, many methods are available to prolong the shelf life of food, including heat treatment, drying, freezing, high pressure, coating, and pickling (Zim et al., 2017). Among them, in the salt or vinegar environment, fermentation pickling with lactic acid bacteria as functional bacteria is a more commonly used preservation method for fruits and vegetables (Mani, 2018). Therefore, if processed properly, the shelf life of red sour soup will be effectively extended (Nout, 1994). Pasteurization is a traditional and relatively mild thermal sterilization method, which can kill pathogenic bacteria and most spoilage bacteria, showing a good effect on inhibiting enzyme activity. To further ameliorate the defect of uneven heating of traditional pasteurization, won II Cho et al. (2016) have changed conduction heating to ohmic heating, and such procedure has been successfully applied in Korean fermented chili sauce. The spore inactivation rate of Bacillus in fermentation broth reaches 99.7%, which effectively improves the shelf‐life of chili sauce. In the field of physical sterilization, in addition to pasteurization, there are also irradiation sterilization technologies using ^60^Co, ^137^Cs, or electron accelerators as irradiation sources. It destroys the DNA of microorganisms in food by high‐energy radiation instead of heating, which is also known as "cold" pasteurization (Ahn et al., 2013; Inabo, 2006). Currently, irradiation sterilization has been widely used in vegetables, fruits, nuts, cereals, meat products, and other agricultural and livestock raw materials because of its terminal sterilization, nonheating, and nonadditive characteristics (Ajibola, 2020). Chemical preservatives (benzoate and sorbate) are used as a type of cost‐efficient storage technology for fermented food. Sodium benzoate and sodium dehydroacetate (SDHA) are often used for the preservation of pickled pepper, and the results show that the expected target can be achieved when the addition of SDHA is 0.03% (Xie et al., 2008). However, it is urgently necessary to develop more reliable methods for food preservation due to the potential microbial resistance and food safety of chemical preservatives (Russell, 1991; Yamazaki et al., 1998). Relatively, the exploitation and application of natural preservatives, such as chitosan, polyphenols, and bacteriocin, have become a new trend in the preservation of fermented food (Delavar & Sedaghat, 2020; Gutiérrez‐del‐Río et al., 2018; Pal, 2018; Santos et al., 2018). Among them, a Lactobacillus metabolite named nisin has been widely used in dairy products, meat products, and seafood because of its excellent safety and significant inhibitory effect on most Gram‐positive food pathogens and spoilage bacteria (Druggan & Iversen, 2014). As one of the characteristic ecological food, the shelf quality of red sour soup has an important impact on the maintenance of brand value. It is of great significance to choose the appropriate technology among various preservation methods to avoid shelf‐life deterioration issues. Based on the abovementioned facts, we aimed to compare the effects of pasteurization, irradiation sterilization, SDHA, and nisin on the microflora and sensory quality of red sour soup after storage. Collectively, our findings provided technical support for shelf‐life quality optimization of red sour soup.

## 2 MATERIALS AND METHODS

### 2.1 Materials and chemicals

Red sour soup was collected from Jinsha Guan Xiang Fang Seasoning Food Co., Ltd. (Guizhou, China). SDHA, nisin, L‐lactic acid (standard product), and glacial acetic acid (standard product) were purchased from Shanghai Aladdin Biochemical Technology Co., Ltd. Activated carbon, ammonium dihydrogen phosphate, and phosphoric acid were obtained from Chengdu Jinshan Chemical Reagents Co., Ltd. Methanol (HPLC grade), E.Z.N.A™ Mag‐Bind Soil DNA Kit, Qubit3.0 DNA reagent test kit, AxyPrepDNA, 2×Hieff® Robust PCR Master Mix, and Hieff NGS™ DNA Selection Beads were provided by Shanghai Macklin Biochemical Technology Co., Ltd., American Omega Bio‐Tek Company, American Life Technologies Company, Aisijin Biotechnology (Hangzhou) Co., Ltd., and Shanghai Yizhong Biotechnology Co., Ltd., respectively.

### 2.2 Sample preparation

The fermented red sour soup (pH =3.7 ± 0.2) was divided into five groups as follows: blank control group (RCK), pasteurization group (RP), irradiation sterilization group (RI), nisin antibacterial group (RN), and SDHA treatment group (RD). Among them, the treatment time of pasteurization was 20 min, the central temperature of samples was set at 65℃, 75℃, 85℃, and 95℃, and the irradiation dose of irradiation sterilization was set at 2 kGy, 4 kGy, 6 kGy, and 8 kGy. The radiation dose of the sample was determined by silver dichromate, and the dose absorption rate was less than 7.5 kGy·S^‐1^. The additive dose in the RN and RD groups was 10, 50, 100, 150, and 200 mg/500 g. The shelf life of samples treated by the optimized conditions was tested for 5 weeks. During the experimental period, the samples were placed in glass sample bottles and stored at room temperature. The colony count in samples was detected according to GB 4,789.2 (CFDA, 2016).

### 2.3 Illumina miseq sequencing

Microbial DNA extraction and PCR amplification were carried out as previously described with minor modifications (Xie et al., 2018). Briefly, 100 ml sample was centrifuged at 4,000 rpm for 5 min, and the precipitate was collected. Total DNA of the samples was isolated using E.Z.N.A™ Mag‐Bind Soil DNA Kit (Omega Bio‐Tek, America) and then sent to Sangon Biotech (Shanghai) Co., Ltd., for sequencing. During the experiment, the PCR amplification was completed in two rounds. In the first round of amplification, after the genomic DNA was accurately quantified using Qubit3.0 dsDNA detection kit (Life Technologies, America), the amount of DNA that should be added to the PCR was determined. The primers used in PCR were fused with part of the linker sequence of the sequencing platform, and the target amplification regions were the V3‐V4 and ITS1‐ITS2 regions of the 16S rRNA gene. Among them, the amplification primers for the V3‐V4 region were 341F (CCTACGGGNGGCWGCAG) and 805R (GACTACHVGGGTATCTAATCC); and the ITS region primers were ITS1F (CTTGGTCATTTAGAGGAAGTAA) and ITS2R (GCTGCGTTCTTCATCGATGC). The PCR product was subjected to 2% agarose gel electrophoresis to confirm whether the length of the amplicons was correct. Then, Agencourt AMPure XP magnetic beads (Aisijin Biotechnology, Hangzhou) were used to recover the target strips. Qubit3.0 dsDNA detection kit was used for detection and quantification, and the libraries were mixed at an equimolar ratio. The library was sequenced using the Illumina MiSeq® platform (Illumina, America), and the sequencing mode was 300 bp at both ends.

### 2.4 Electronic nose analysis

An electronic nose (Airsence PEN3, Germany) was used to analyze the odor difference of samples preserved under different conditions. Briefly, 1.0 g sample was accurately weighed and placed in a headspace bottle, followed by incubation in a water bath at 60 ℃ for 20 min. Odor fingerprint analysis conditions were set as follows: the sampling interval was 1 s, the sensor self‐cleaning time was 40 s, the sensor zeroing time was 10 s, the sample preparation time was 5 s, the sampling analysis time was 120 s, and the injection flow rate was 400 ml/min.

### 2.5 Total acid and organic acid analysis

The analysis of total acid in the red sour soup was carried out according to T/CNFIA 117 (CNFIA, 2020) and GB/T 12,456 (SAC, 2008). The analysis of organic acid was conducted using liquid chromatography as previously proposed (Xiong et al., 2012).

### 2.6 Sensory evaluation

The color, smell, taste, and tissue state of red sour soup were investigated according to the requirements of "sour soup seasoning" (CNFIA, 2020). Briefly, 10 experimental personnel were invited to participate in the sensory evaluation, and all participants were trained according to the relevant provisions of GB/T 16,291.1 (SAC, 2012). Sensory evaluation was conducted by a scoring system. The full score range of color was 30, and a score of 0–10, 10–20, and 20–30 indicated poor, fair, and well, respectively. The full score range of smell and taste was 40, and a score of 0–20, 20–30, and 30–40 indicated poor, fair, and well, respectively. The full score range of tissue state was 30, and a score of 0–10, 10–20, and 20–30 indicated poor, fair, and well, respectively.

### 2.7 Rheological property analysis

To study the rheological properties of samples preserved under different conditions, the rheologic behavior of red sour soup was analyzed by rheometer (DHR‐1, TA Instruments, America), including static shear test and rheological behavior in oscillatory mode. For the static shear test, the apparent viscosity and shear stress of red sour soup were measured at 25 ℃ with a shear rate ranging from 0.1 s^‐1^ to 100 s^‐1^. The Ostwald model was used to assess shear rate (x) and shear stress (*y*), and flow index (*n*), consistency coefficient (*k*), and complex correlation coefficient *R^2^* were recorded. Ostwald equation: *y* = *kx^n^* (1). Rheological behavior was tested in the oscillatory mode. A plate mold with a diameter of 60 mm was selected, the gap was 1.0 mm, the angular frequency was 1 Hz, and the stress was 5.00 Pa. The red sour soup was placed on the measuring platform, and the cover plate was pressed down. The storage modulus of red sour soup was tested at the temperatures programmed from 25 ℃ to 90 ℃ and then from 90 ℃ to 25 ℃ for 1 min at a rate of 5 ℃/ min.

### 2.8 Data analysis

The data were presented as mean ±standard deviation (*SD*), and the significant differences among the groups were analyzed by SPSS17.0 software with 95% confidence. The results of Illumina MiSeq sequencing were analyzed by Cutadapt (version 1.18), Pear (version 0.9.8), Prinseq (version 0.20.4), Usearch (version 11.0.667), RDP classifier (version 2.12), NCBI Blast+ (version 2.10.0), R (version 2.5–6), Ggraph (version 2.0.0), Gplots (version 3.0.1.1), VennDiagram (version 1.6.20), and UpSetR (version 1.4.0).

## 3 RESULTS AND DISCUSSION

### 3.1 Single‐factor optimization for different preservation methods

**FIGURE 1:**
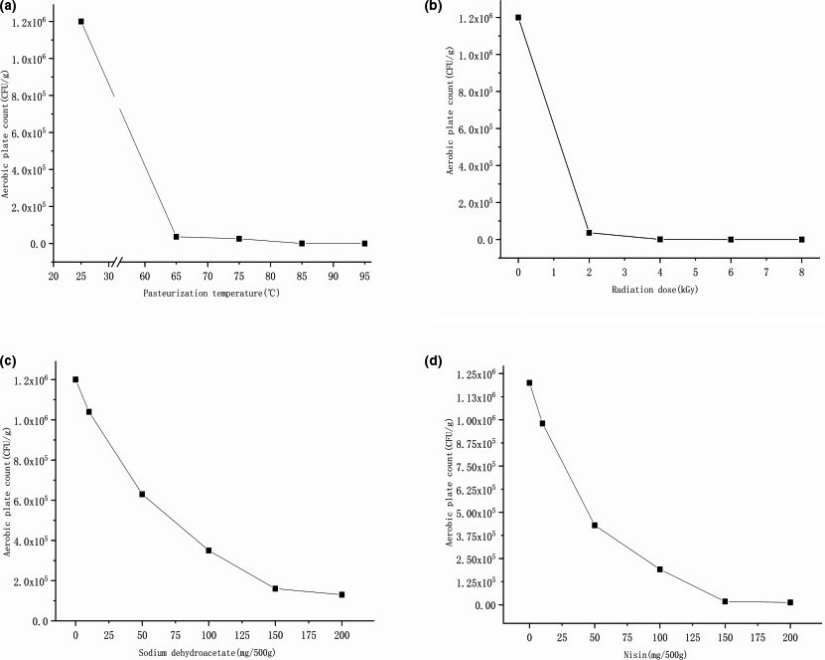
Effects of different preservation methods on the colony count in red sour soup. (a) describes the change of the aerobic plate count in the sample with the increase of pasteurization temperature; (b) describes the change of the aerobic plate count in the sample with the increase of irradiation dose; (c) describes the change of the aerobic plate count in the sample with the increase of SDHA dosage; (d) describes the change of the aerobic plate count in the sample with the increase of nisin dosage

Before we investigated the effects of pasteurization, irradiation, SDHA, and nisin on the shelf quality of red sour soup, the heating temperature, irradiation dose, and dosage of preservatives were optimized by single‐factor experiments, and the results were shown in Figure 1. The colony count was 1.2 × 10^6^ CFU/g in the red sour soup without preservation treatment, while the total bacteria changed dramatically after thermal, such as pasteurization, and nonthermal (irradiation, SDHA, nisin) processing technology. For pasteurization, the sterilization temperature was negatively correlated with the colony count. When the temperature reached 85 ℃, the colony count in the sample was only 200 ± 10 CFU/g and there was no significant change in the sensory quality of the samples. The results of the single‐factor experiment also showed that the colony count of the red sour soup was sharply decreased when the irradiation dose was more than 2 kGy. At 4 kGy, the colony count was less than 1,000 CFU/g, reaching 900 ± 43.59 CFU/g. Our results were consistent with previous studies that most pathogenic microorganisms in food are killed when the irradiation dose is 1–10 kGy (Morehouse & Komolprasert, 2004). BPOM (2006) has also pointed out that the radiation dose below 10 kGy is harmless, and there is no need to carry out relevant toxicological experiments. The associations between the total colony count and the dose of SDHA or nisin showed the same trend, while the antibacterial effect of the latter was significantly better compared with the former. When the dose was 150 mg/500 g, the colony count in the RD group was 1.6 × 10^5^ CFU/g, and the colony count in the RN group was 1.8 × 10^4^ CFU/g. As the dose was increased, the total colony count changed insignificantly. Similarly, the dose and effect of SDHA and nisin have also been proved in the studies of Xie et al. (2008) and Chang et al. (2019). Therefore, the temperature of the RP group was set to 85℃, the irradiation dose of the RI group was limited to 4 kGy, and the additive dose of SDHA and nisin of the RP and RD groups was 150 mg/500 g in the experiment. Subsequently, a 5‐week shelf‐life experiment was carried out on the red sour soup samples with different treatments. The results showed that the colony count in all treated groups was much lower compared with the RCK group during the experimental period. The RD group and RCK group showed an upward trend in the total colony count at the 3rd week, reaching 8.2 × 10^5^ CFU/g and 7.4 × 10^6^ CFU/g at the 5th week, respectively.

### 3.2 Microflora analysis of samples under different preservation conditions

**FIGURE 2:**
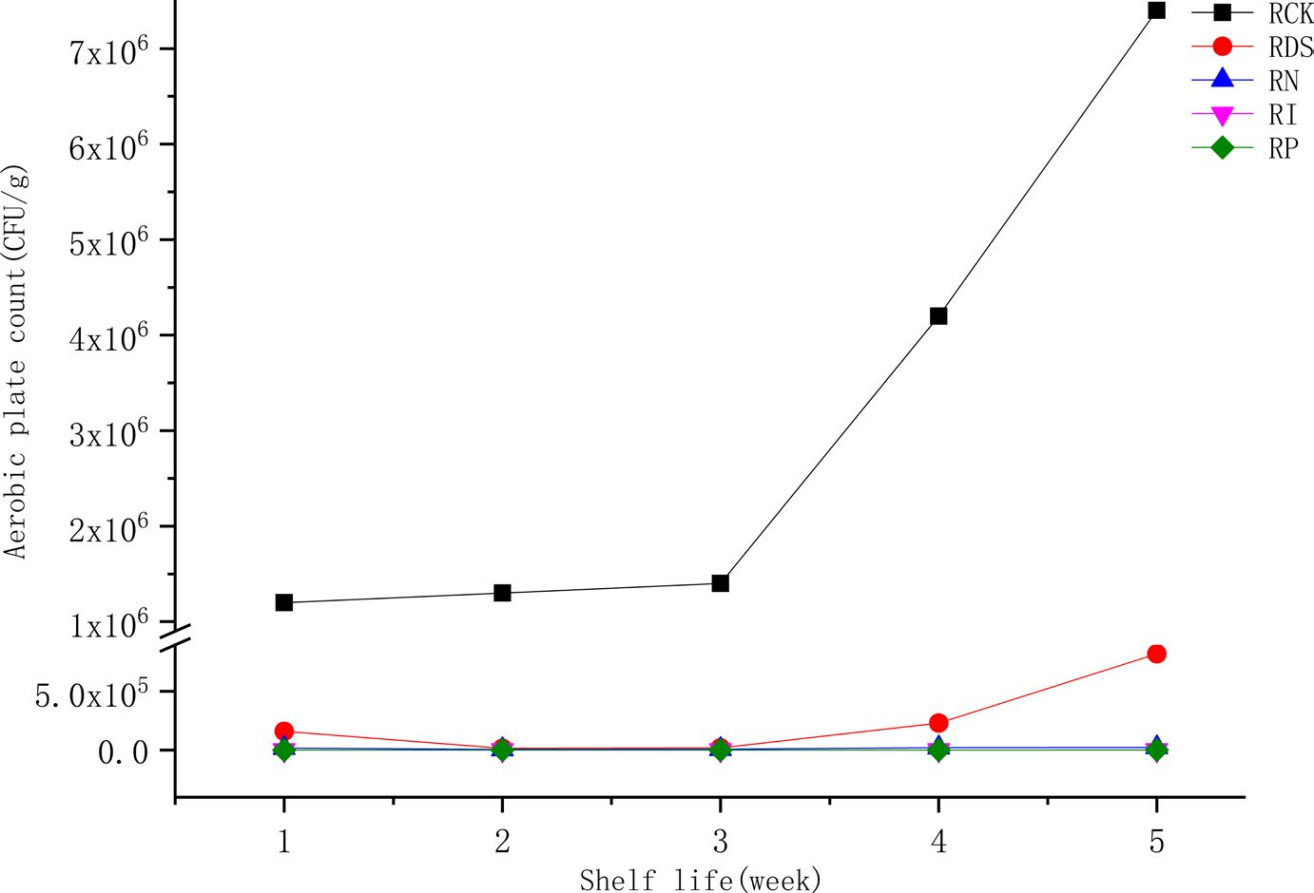
The changing trend of the colony count with shelf life in red sour soup under different preservation conditions

**FIGURE 3:**
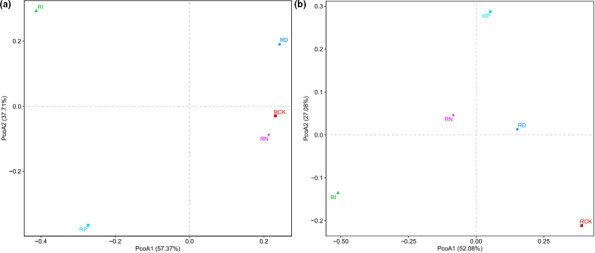
PCoA chart of microbial species composition in red sour soup with different treatments. (a) is for bacteria; (b) is for fungi

**FIGURE 4:**
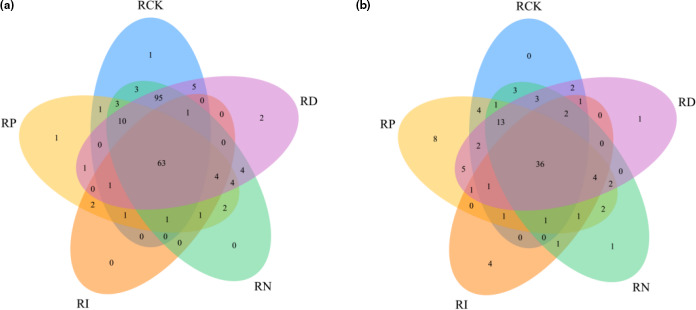
Venn diagram of species distribution among samples based on the OTU number. (a) is for bacteria; (b) is for fungi

**FIGURE 5:**
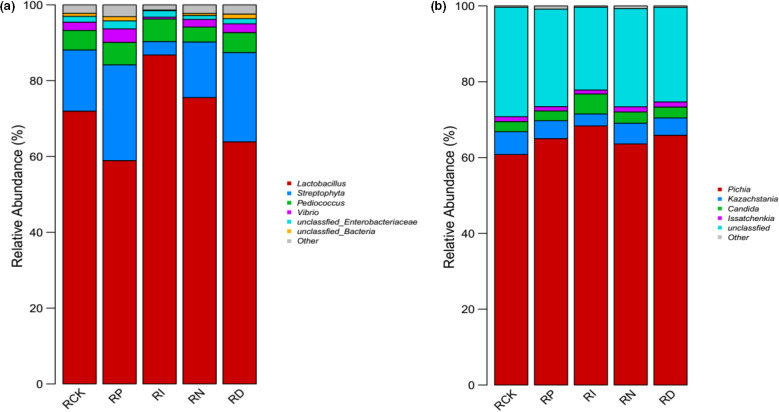
Relative abundance histogram at the genus level. (a) Describes the composition of the bacteria in the sample; (b) describes the composition of the fungi in the sample

To clarify the composition of microbial flora in red sour soup after 5 weeks of storage, Illumina MiSeq sequencing with high‐throughput, parallel, and quantitative characteristics was used to determine and analyze the experimental and blank control samples (Zhai et al., 2016). The Chao and Shannon indices were used to evaluate the microbial diversity in the samples. Table 1 shows that the microbial diversity of the RI group and RP group was the lowest among all samples (Figure 2). On the other hand, the bacterial diversity of the RN group and RD group was similar to the RCK group, while the abundance of fungal species was between the RI group and RP group. Besides, to further verify the distribution of microbial species in the samples, the principal coordinate analysis (PCoA) in the β‐diversity analysis was also used for auxiliary explanation, and detailed information was given in Figure 3. The Venn diagram depicted the common or unique species classification in different treatment groups. The total operational taxonomic unit (OTU) number of bacteria and fungi was 206 and 100, respectively. The number of OTUs shared by bacteria was 63, and that shared by fungi was 36. The histogram of relative abundance of dominant species showed that *lactobacillus*, *streptomyces*, and *pediococcus* were the three most important genera of bacteria in different treatments, and *pichia*, *kazachstania*, and *candida* were the most important fungi affecting the quality of red sour soup (Figures 4, 5). The dominant microorganism in our study was similar to previous studies (Lin et al., 2020; Wang et al., 2020). In comparison, the relative abundance of *lactobacillus* and *pichia* was higher in the RI group, indicating a stronger killing ability than other microorganisms. However, the excessive reproduction of *lactobacillus* and *pichia* will also lead to the occurrence of postacidification, gas production, mildew, and other adverse events. Therefore, it is necessary to further clarify how to properly sterilize the product (Zhang, 2018).

**TABLE 1:** Analysis of community richness and *α*‐diversity in different groups of red sour soup

Sample	Target	Number	OTUs	Chao	Shannon	Coverage
RCK	16S r DNA	59,526	185	195.22	2.00	1.00
	ITS	124,573	70	107.50	1.23	1.00
RP	16S r DNA	68,407	95	99.00	1.24	1.00
	ITS	68,839	82	88.00	1.18	1.00
RI	16S r DNA	63,477	74	79.00	1.58	1.00
	ITS	62,986	53	61.25	1.11	1.00
RD	16S r DNA	75,489	190	195.45	2.00	1.00
	ITS	170,101	73	76.00	1.13	1.00
RN	16S r DNA	74,474	191	197.79	1.87	1.00
	ITS	137,772	70	79.55	1.21	1.00

Note

The umber means number of sequences of samples, OTUs means the number of OTUs obtained by sample clustering, and Chao and Shannon means the value of diversity index type in each sample.

Abbreviations: RCK, Red sour soup control check group; RD, Red sour soup SDHA group; RI, Red sour soup irradiation group; RN, Red sour soup nisin group; RP, Red sour soup pasteurization group.

### 3.3 Effects of different preservation methods on the flavor of red sour soup

**FIGURE 6:**
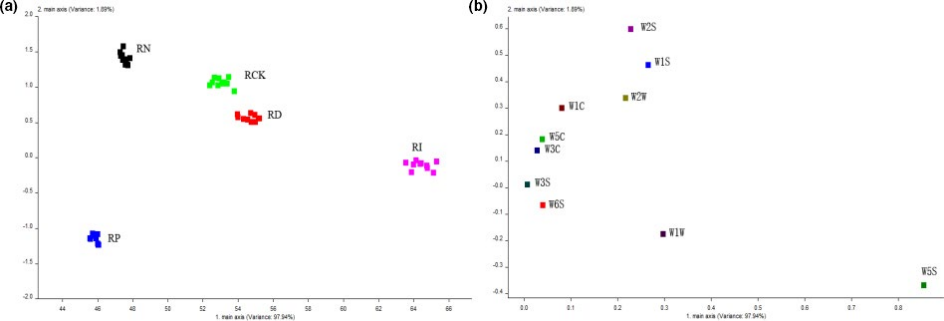
Electronic nose analysis of red sour soup under different preservation conditions. (a) is PCA analysis, and (b) is LoA analysis

**FIGURE 7:**
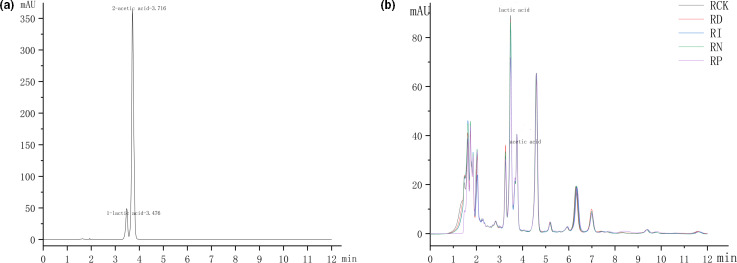
the chromatogram of HPLC. (a) is the standard chromatograms for lactic acid and acetic acid; (b) is the chromatograms of samples under different preservation conditions

**FIGURE 8:**
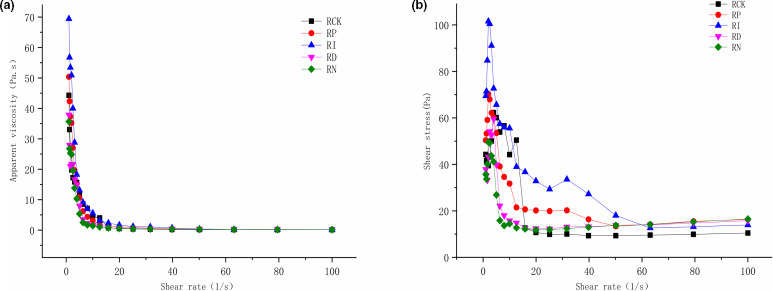
Effect of different preservation treatments on apparent viscosity and shear stress for red sour soup. (a) and (b) represent the changing trend of apparent viscosity and shear stress with the shear rate in different samples, respectively

**FIGURE 9:**
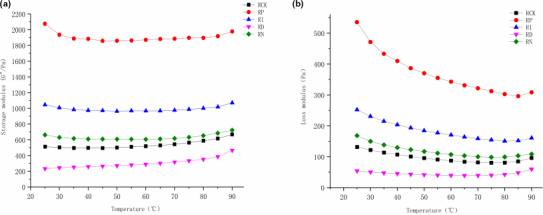
Changes of storage modulus and loss modulus by different preservation treatments in the heating process. (a) and (b) represent the changing trend of storage modulus and loss modulus with the temperature in different samples, respectively

As a seasoning, the flavor of the red sour soup is directly affected by the smell and taste. Preservation methods not only affect the distribution of microbial communities but also impact the flavor and rheological properties of the products. To minimize the experimental error caused by human factors, electronic nose technology, total acid titration, and liquid chromatography were used to test and analyze the differences in odor and sour taste of differently processed samples. The PCA results of electronic nose showed that the first and second principal components contributed 97.94% and 1.89% to the odor of red sour soup, respectively, and the total contribution rate of the two principal components was 99.83%, basically representing the main information characteristics of five differently processed samples. In the first principal component, the flavor of RN, RP, and RI groups was significantly different from the RCK group, and there were also significant differences among RN, RP, and RI groups. In the second principal component, the RI and RP groups were still significantly different from the RCK group. The limits of agreement (LoA) analysis showed the response of the electronic nose sensor to 10 types of odor components in red sour soup. Among them, the components detected by five sensors, W1W (sulfide), W2W (organic sulfide), W1S (methyl), W2S (alcohols, ketones, and aldehydes), and W5S (nitrogen oxides) significantly contributed to the flavor difference of different treatments. The results of titration and liquid phase analysis showed that there was no significant difference in the content of total acid in the samples preserved under different conditions. However, the contents of organic acids, such as lactic acid and acetic acid, in the RI and RP groups were slightly lower compared with the RN, RD, and RCK groups. Sensory evaluation showed that there were no significant differences in color, smell, and taste among other groups, except that the tissue state score of the RP group was lower. The abovementioned results were shown in Figures 6, 7 and Table 2, 3. All of the results showed that pasteurization and irradiation treatment affected electronic nose data and organic acid content in red sour soup. The sensory changes of the pasteurization group might be attributed to the loss of volatile components caused by heating, while the differences of the irradiation group might be attributed to sulfur, methyl, and ketaldehyde compounds produced in the process (Yoo et al., 2003). Organic acid is the main contributor to the special flavor of red sour soup. We found that the total acid content in each treatment group was not significantly different. However, the content of lactic acid in each treatment group was lower compared with the RCK group, and the RI group showed the lowest content. This finding might be attributed to the inhibitory effect of preservation technology on the growth of lactic acid bacteria, resulting in the retarded postacidification (Figures 8, 9).

**TABLE 2:** Contents of total acids and organic acids in red sour soup under different preservation conditions

Sample	Total acids (g/100 g)	Lactic acid (mg/mL)	Acetic acid (mg/mL)
RCK	2.51 ± 0.34^a^	1.83 ± 0.04^a^	0.75 ± 0.03^a^
RP	2.47 ± 0.20^a^	1.41 ± 0.10^b^	0.73 ± 0.09^a^
RI	2.54 ± 0.53^a^	1.73 ± 0.02^a^	0.69 ± 0.03^a^
RD	2.52 ± 0.24^a^	1.76 ± 0.23^a^	0.74 ± 0.06^a^
RN	2.50 ± 0.37^a^	1.74 ± 0.16^a^	0.73 ± 0.12^a^

Notes

The values are displayed as means±*SD*. Different letters in the same column showed a significant difference between groups (*p* <.05).

Abbreviations: RCK, Red sour soup control check group; RD, Red sour soup SDHA group; RI, Red sour soup irradiation group; RN, Red sour soup nisin group; RP, Red sour soup pasteurization group.

**TABLE 3:** Sensory evaluation of red sour soup under different preservation conditions

Sample	Color	Smell and taste	Texture
RCK	29.2 ± 0.84^a^	38.6 ± 1.47^a^	27.8 ± 1.92^a^
RP	28.6 ± 1.35^a^	37.9 ± 2.25^a^	23.6 ± 2.30^b^
RI	28.9 ± 0.82^a^	37.6 ± 1.71^a^	27.1 ± 1.95^a^
RD	28.9 ± 0.65^a^	38.7 ± 0.91^a^	27.6 ± 1.62^a^
RN	28.5 ± 0.45^a^	38.4 ± 1.08^a^	27.4 ± 2.07^a^

Notes

The values are displayed as means±*SD*. Different letters in the same column showed significant difference between groups (*p* <.05).

Abbreviations: RCK, Red sour soup control check group; RD, Red sour soup SDHA group; RI, Red sour soup irradiation group; RN, Red sour soup nisin group; RP, Red sour soup pasteurization group.

### 3.4 Rheological properties of red sour soup under different preservation conditions

Rheological data play an important role in the analysis of flow conditions in food processing, such as pasteurization and aseptic processing (Michèle et al., 2001). To further evaluate the effects of different preservation methods on the quality of red sour soup, two indications of apparent viscosity and thixotropy were introduced. The viscosity was evaluated by the effect of shear rate on the apparent viscosity, and the thixotropy was determined based on the thixotropy and temperature scanning. The results showed that the apparent viscosity of red sour soup was decreased with the increase or decrease in shear rate, and it tended to be stable after the shear rate reached 20 (1/s). The apparent viscosity showed a trend of RI <RP < RCK <RD < RN, while the difference among RCK, RD, and RN groups was not significant. The shear stress was increased first and then decreased with the shear rate. RCK, RD, and RN groups tended to be stable after the shear rate reached 20 (1/s), while RI and RP groups were gradually balanced at 50 (1/s), indicating that irradiation and pasteurization had effects on the thixotropic properties of red sour soup. Besides, the temperature scanning results showed that the storage modulus of the samples was relatively flat with the increase in temperature, while the loss modulus showed a decreasing trend. In the whole temperature range, the tanδ of each sample was less than 1, indicating that all samples had weak gel properties. When the temperature was greater than 60℃, the tanδ value of RCK and RD groups was less than that of RI and RP groups, indicating that the gel properties of RI and RP groups were more seriously weakened at a higher temperature. This might be attributed to the degradation of pectin in chili, tomato, and other raw materials in red sour soup by irradiation and heat treatment (Dogan and Kayacier, 2007; Jiang et al., 2019).

## 4 CONCLUSIONS

Overall, pasteurization (85 ℃, 20 min), irradiation sterilization (4 kGy), SDHA (150 mg/500 g), and nisin (150 mg/500 g) had positive effects on guaranteeing the shelf life quality of the red sour soup. Among them, the preservation effect of irradiation and pasteurization was comparable to chemical and natural additives. *Lactic acid bacteria* and *Pichia* were the dominant microorganisms in red sour soup, which might be the important reasons for gas production in the later stage. Besides, irradiation could affect the odor, acidity, and rheological properties of red sour soup. However, these changes had no significant effects on the sensory quality of the product. Therefore, irradiation sterilization could be used as an ideal quality preservation method for red sour soup seasoning. The preservation effects of physical cold sterilization and natural additives need to be further explored.

## AUTHOR CONTRIBUTIONS

**Yongfu Li:** Supervision (supporting); Validation (equal). **Xingbang Luo:** Conceptualization (equal); Project administration (supporting); Resources (supporting). **Guolin Li:** Formal analysis (supporting); Software (supporting). **Zhaoyan Duan:** Data curation (equal); Investigation (equal); Methodology (supporting). **Chaojun Chen:** Data curation (equal); Investigation (equal); Methodology (equal).
